# A Comprehensive Review of Pedunculagin: Sources, Chemistry, Biological and Pharmacological Insights

**DOI:** 10.3390/ijms252111511

**Published:** 2024-10-26

**Authors:** Julia Snarska, Katarzyna Jakimiuk, Jakub W. Strawa, Tomasz M. Tomczyk, Monika Tomczykowa, Jakub P. Piwowarski, Michał Tomczyk

**Affiliations:** 1 Student’s Scientific Association, Department of Pharmacognosy, Faculty of Pharmacy with the Division of Laboratory Medicine, Medical University of Białystok, ul. Mickiewicza 2a, 15-230 Białystok, Poland; julia.snarska.js@gmail.com (J.S.); 43287@student.umb.edu.pl (T.M.T.); 2 Department of Pharmacognosy, Faculty of Pharmacy with the Division of Laboratory Medicine, Medical University of Białystok, ul. Mickiewicza 2a, 15-230 Białystok, Poland; katarzyna.jakimiuk@umb.edu.pl (K.J.); jakub.strawa@umb.edu.pl (J.W.S.); 3 Department of Organic Chemistry, Faculty of Medicine with the Division of Dentistry and Division of Medical Education in English, Medical University of Białystok, ul. Mickiewicza 2a, 15-222 Białystok, Poland; monika.tomczyk@umb.edu.pl; 4 Microbiota Lab, Department of Pharmaceutical Biology, Medical University of Warsaw, ul. Banacha 1, 02-097 Warsaw, Poland; jakub.piwowarski@wum.edu.pl

**Keywords:** tannins, pedunculagin, chemistry, pharmaceutical analysis, biological activity

## Abstract

Pedunculagin is a widely abundant ellagitannin found in the plant kingdom, with a chemical structure featuring two hexahydroxydiphenoyl units linked to a glucose core. It has demonstrated various biological activities, including anti-cancer, anti-inflammatory, and anti-bacterial effects. This review aims to summarize the bioactivities, chemistry, and health-promoting properties of pedunculagin and plant preparations containing it. It is the first comprehensive summary covering pedunculagin’s chemistry, sources, metabolism, and other relevant research. The search databases were Google Scholar, EBSCO Discovery Service, REAXYS Database, SCILIT, SCOPUS, PubMed, MEDLINE, Web of Science, Wiley Online Library, Science Direct/ELSEVIER, WordCat, and Taylor and Francis Online. All the databases were methodically searched for data published from 1911 until 2024. Various biological effects were proven in vitro for pedunculagin; however, due to the limited availability of the isolated compound, they have not been so far directly confirmed on more advanced in vivo and clinical models. However, its bioactivity can be deduced from studies conducted for plant preparations containing this ellagitannin as a dominant constituent, consequently indicating beneficial health effects. Further studies are needed to determine the molecular mechanism of action following topical application as well as the contribution of gut microbiota postbiotic metabolites– urolithins–being formed following the oral ingestion of preparations containing pedunculagin.

## 1. Introduction

Tannins (hydrolyzable and non-hydrolyzable/condensed) are nitrogen-free natural substances with a high molecular weight, are soluble in water, have the nature of polyphenols, and contain numerous hydroxyl groups. Tannins can create permanent connections with proteins and polysaccharides, glycoproteins, and glycolipids, modifying their spatial structure and thus changing their biological properties. When used skillfully, even in low concentrations—without tannins—they influence the spatial structure and biological properties of molecules that play key roles in the biochemical processes induced by receptors and their ligands, toxins, and xenobiotics [1]. Skillfully used active tannins can stop the vicious circle of the pathology of many chronic inflammations of the mucous membranes of the digestive tract, respiratory system, urinary tract, reproductive tract, and skin. Tannins can help in the effective prevention and treatment of lifestyle diseases by binding environmental toxins consumed with food, reducing their multiplication and limiting the destructive impact on the intestinal mucosa—pathogenic microorganisms in the increasingly observed intestinal dysbiosis. Tannins can maintain the tightness of mucous membranes, ensure the proper presentation of environmental antigens, and the function of the MALT system and the entire immune system and other adaptogenic systems (nervous, circulatory, endocrine). Tannins used prophylactically—in combination with prebiotics—can protect us against traveler’s diarrhea, destructive gastrointestinal dysbiosis and its consequences (endotoxemia), chronic inflammation leading to lifestyle diseases (atherosclerosis, cancer, autoimmune diseases), allergies, metabolic disorders, and dysfunction of adaptogenic systems [2,3]. The tangible goal of our study is to present all the comprehensive facts about the beneficial health-promoting properties of one of the hydrolyzable tannins, pedunculagin.

Despite the fact that the different bioactivities of pedunculagin have been established, there are no distinctly organized review articles available. This is the first such comprehensive summary describing the molecule pedunculagin and its chemistry, sources of occurrence, metabolism, and analytical and biological research to showcase its potential to be used as a therapeutic agent.

## 2. Research Methodology

This annotated bibliography focuses on the various approaches to studying pedunculagin. First, the search databases for this review were defined to search records for the main keyword “pedunculagin” to ensure the inclusiveness of the most relevant publications. We conducted electronic searches in the following databases (n = numbers of records/documents): Google Scholar (n = 3610), EBSCO Discovery Service (n = 2407), REAXYS Database (n = 471), SCILIT (n = 183), SCOPUS (n = 242), PubMed/MEDLINE (n = 109), Web of Science (n = 159), Wiley Online Library (n = 245), Science Direct/ELSEVIER (n = 652), WordCat (n = 80), and Taylor and Francis Online (n = 111). All the databases were methodically searched for publications, abstracts, conference papers, and books published from 1911 until 2024.

Suitable records/publications (originals, reviews, conference abstracts, books) were manually chosen from the following searches: pedunculagin or pedunculagin AND other keywords: chemistry, biological activity, biological potential, occurrence, biosynthesis, bio-inspired synthesis, organic synthesis, chromatography, analysis, isolation, identification, nuclear magnetic resonance, traditional use, pharmacological, natural product, secondary metabolites, therapeutic agent, quantitative analysis, qualitative analysis, antitumor, anticancer, antifungal, or antioxidant.

The chemical structure was drawn on the online Chem Draw software (http://chemdrawdirect-cdn.perkinelmer.com). URL (accessed on 1 September 2024). The physicochemical properties were predicted by the ACD/Labs Percepta Platform (Toronto, ON, Canada) and Chemaxon (Budapest, Hungary).

## 3. The Occurrence of Pedunculagin in the Plant Kingdom

Ellagitannins are commonly found in plants of the Rosaceae family as well as in some nuts, seeds, and fruits. Their content has been best documented with regard to raspberries/blackberries (*Rubus* L., Rosaceae). Some species of the *Rubus* L. genus such as cloudberry (*R. chamaemorus*) and raspberry *(R. idaeus*) have been tested for the presence of pedunculagin. High concentrations of pedunculagin have also been observed in extracts obtained from common walnut (*Juglans regia*, Juglandaceae) [4,5]. Pedunculagin-containing plant species are summarized in Table 1.

## 4. Chemical Structure of Pedunculagin

**Figure 1:**
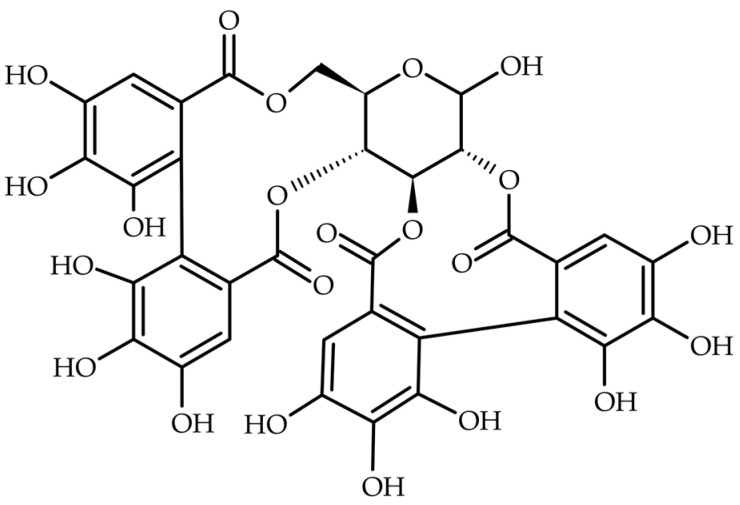
Structural formula of pedunculagin.

Pedunculagin (Figure 1) belongs to the class of ellagitannins (hydrolyzable tannin)—a broad class of compounds, more than 500 of which have been identified to date. The high variability and diversity of this class are due to the structural presence of two distinct components capable of binding with one another in any configuration [101,102]. These consist of a hexahydroxydiphenyl acid bond being formed between two gallic acid molecules and a monosaccharide, which is usually D–glucose [103]. In the typical ester structure of pedunculagin, two HHDP acid residues can be distinguished, connected to the hydroxyl groups at the carbons in positions 2,3– and 4,6– of the D–glucopyranose molecule, respectively [104]. The molar mass of pedunculagin is 784.5 g/mol, and the compound is represented by the molecular formula C_34_H_24_O_22_. Pedunculagin also goes by the names cyclic 2,3:4,6–bis(4,4′,5,5′,6,6′–hexahydroxydiphenate)–alpha–D–glucopyranose or cyclic 2,3:4,6–bis(4,4′,5,5′,6,6′–hexahydroxydiphenate)–beta–D–glucopyranose, depending on its anomeric form. The compound has the specific rotation [α]^25^_578_ of +106° in MeOH, and the pH values of its aqueous solutions range from 4.7 to 4.9 [105].

Considering incomplete experimental data, the predicted physicochemical properties of pedunculagin are as follows: density 1.92 g/mL; theoretical log P = 1.95; log D (pH 7.4) = 0.98; strongest acidic pKa = 6.86; pH-dependent solubility (logS) = −3.61; water solubility = 8.34 × 10^−2^ mol/L; boiling point 1578 ± 65 °C at 760 mmHg; melting points = 196 °C; hydrogen bond donor (HBD) = 13 hydrogen bond acceptor (HBA) = 22, index of refraction = 1.79. Other properties of pedunculagin are as follows: optical rotatory power [α]_D_ (c = 1.1 g/100 mL methanol, 589 nm, 25 °C) = 88–89 degree, CD (MeOH) 238 nm, +41.9, 263 nm, −14.7, 284 nm, +6.5; MS (FAB-) m/e785.3 (MD+) [10,101].

## 5. Organic Synthesis of Pedunculagin

In the process of designing the synthesis of biaryl compounds, the different regiochemical and stereochemical properties of potential products need to be taken into account. Schmidt and Haslam observed that ellagitannins with HHDP moieties at positions 2,3 and 4,6 of the glucose core occur mainly in the form of (S) atropoisomers. On this basis, it was inferred that the final product of the synthesis is most influenced by the conformational preference of the sugar residue, i.e., glucose. The formation of a particular diastereoisomer is closely related to the amount of energy required to form the transition state system in the course of the process. At the bond formation stage, less energy needs to be supplied to the precursor of the (S) conformer than to the precursor of the (R) conformer. As a result, ellagitannins are predominantly observed in their enantiomeric (S) forms [105,106]. Ellagitannin synthesis can be carried out in two ways, which differ significantly in their yields. The first involves intramolecular oxidative coupling of phenolic aromatic compounds linked to D–glucose. In spite of the sugar core’s preference for the attachment of subunits at the O(4) and O(6) positions, a better method, leading to higher yields, involves these subunits being attached to the O(2) and O(3) positions first. Obtaining pedunculagin requires gallic acid molecules to form bonds between two carbon atoms of neighboring molecules, yielding hexahydroxydiphenyl acid (HHDP) moieties. For this purpose, after esterification of the sugar moiety with gallic acid derivatives under standard conditions, a higher dilution and lower temperature are required compared to the coupling of subunits at positions C-4 and C-6. Then, with the mediation of Pb(OAc)_4_, the carbons of the sugar-bound galloyl residues form a bond, leading to the formation of one of the two HHDP groupings present in the structure of pedunculagin. The incorporation of the second HHDP moiety is achieved in an analogous manner. After purification, a 1:1 mixture of *α* and *β* anomers is obtained [105]. In contrast, the second pathway involves the attachment of HHDP moieties to the D–glucose molecule by double intramolecular esterification. The simultaneous and direct attachment of two residues of (S)–hexabenzyloxydiphenyl acid at both the 2,3 and 4,6 positions facilitates a significant reduction in the number of synthetic steps, thus accelerating the entire process and, at the same time, increasing the quantity of the product obtained [104,107].

## 6. NMR and Mass Spectral Characterization of Pedunculagin

Pedunculagin is a light-brown amorphous powder containing a mixture of *α* and *β* anomers. As shown by ESI–TOF MS analysis, the *m/z* ratio of pedunculagin following negative ionization is 783.0671 [M–H]^−^, translating to the molecular formula of C_34_H_23_O_22_ (783.0686). The chemical shifts as observed in the nuclear magnetic resonance analysis of pedunculagin are as follows:

^1^H NMR (*acetone–d*_6_, 500 MHz), δ: 3.79 (1H, dd, *J* = 13.2 Hz, H–6α), 3.85 (1H, dd, *J* = 13.1 Hz, H–6β), 4.22 (1H, dd, *J* = 10.6 Hz, H–5β), 4.61 (1H, dd, *J* = 10.7 Hz, H–5α), 4.86 (1H, dd, *J* = 9.8 Hz, H–2β), 5.06 (1H, d, *J* = 8 Hz, H–1β), 5.07 (1H, dd, *J* = 10.4 Hz, H–2α), 5.08 (1H, t, *J* = 10 Hz, H–4α), 5.08 (1H, t, *J* = 10 Hz, H–4β), 5.24 (1H, dd, *J* = 10.9 Hz, H–3β), 5.26 (1H, dd, *J* = 13.7 Hz, H–6α), 5.30 (1H, dd, *J* = 13.6 Hz, H–6β), 5.46 (1H, d, *J* = 4, H–1α), 5.47 (1H, t, *J* = 10 Hz, H–3α), 6.33 and 6.52 (2H, s, HHDP–6′′/6′′β), 6.34 and 6.57 (2H, s, HHDP–6′′/6′′α), 6.60 and 6.67 (2H, s, HHDP–6′′/6′′β), 6.61 and 6.66 (2H, s, HHDP–6′′/6′′α).
^13^C NMR (*acetone–d*_6_, 125 MHz), δ: 63.6 (2C, C–6α/β), 67.5 (C–5α), 69.7 (C–4β), 69.9 (C–4α), 72.6 (C–5β), 75.6 (C–2α), 75.9 (C–3α), 77.7 (C–3β), 78.5 (C–2β), 91.8 (C–1α), 95.4 (C–1β), 107.3, 107.4, 107.6, 107.7, 107.8, 107.9, 108.4 and 108.5 (8C, HHDP–6′/6′′α/β) [108].

## 7. Methods of Extraction and Isolation of Pedunculagin from Plant Material

To obtain extracts for isolation of pedunculagin, Orabi and co-workers performed defatting of dry *Lawsonia inermis* powdered leaves by repeated steeping in *n*-hexane for consecutive days and homogenization in 70% acetone. After that, they fractionated it on an HP-20 column using a methanol–water solution [61].

High-performance liquid chromatography (HPLC) is a technique enabling the content of compounds of plant origin to be determined. Due to the propensity of tannins to hydrolyze, an important element of the workup is the selection of an appropriate extractant and solvent system for analytical purposes [109]. All the analytical parameters applicable to the analysis of pedunculagin are provided in Table 2 and Table 3. Recent advancements in green chemistry have highlighted deep eutectic solvents (DESs) as an attainable alternative to traditional organic solvents for extracting bioactive compounds, including tannins such as pedunculagin [110]. Sukor et al. applied DESs for onion peel extraction in combination with sonification, where pedunculagin was dominated [111].

## 8. Biological Activity of Pedunculagin

### 8.1. Angiogenesis-Stimulating Effect

An increase in the amount of pro-inflammatory mediators, including cytokines, growth factors, and enzymes that stimulate angiogenesis, was observed as an effect of pedunculagin administration. Pro-inflammatory cytokines, among which TNF–α plays a key role, induce the production of vascular endothelial growth factor (VEGF), which regulates the growth and development of blood vessels and promotes the proliferation, migration, and survival of endothelial cells. To evaluate the ability of pedunculagin to stimulate the formation of new blood vessels, a study was conducted using the chorioallantoic membrane (CAM) of chicken embryos. The results showed that membranes treated with pedunculagin solutions at concentrations of 15 μg/μL and 30 μg/μL showed a significant increase in vascularization compared to the control sample. The average lengths and sizes of individual vessels also increased, with noticeable thickening of the vessels also observed [114].

### 8.2. Anticancer Effects

Pedunculagin causes growth inhibition in adriamycin-resistant human breast cancer cells (MCF–7/Adr) at 10 µM as well as at a higher concentration of 313 μM (43.7% and 73.3% decrease in viability, respectively). The anticancer activity of ellagitannins may be related to the presence of HHDP moieties being released in the form of ellagic acid and its derivatives. The greater the number of these moieties within a molecule, the greater the cancer cell survival inhibition effect. The macromolecular structures do not penetrate cell membranes; their mechanism involves altering the properties of membranes by forming complexes with membrane proteins so that ellagic acid, a product of their hydrolysis, can penetrate into the cell and inhibit cell division. The antitumor effect is a result of the external effect of pedunculagin and the internal activity of its breakdown products [115]. The anticancer properties of pedunculagin have also been attested by the results of studies conducted on mice. Regardless of the dosage, the average survival time of laboratory animals increased by 20.82% relative to the control group [116].

### 8.3. Antioxidant Effect

It is known that tannins, particularly hydrolysable tannins, may act as antioxidant agents. The antioxidant activity of pedunculagin was demonstrated on the basis of the scavenging activity of the stable 2,2–diphenyl–1–picrylhydrazyl (DPPH) free radicals in a manner proportional to its concentration (where IC_50_ = 2.41 ± 0.71 µM) [117]. The compound also demonstrated activity similar to that of superoxide dismutase (SOD), whose action is based on the capture of free radicals. Also, in the extracts with the detection of pedunculagin at elevated concentrations, strong antioxidants were also observed [118].

### 8.4. Stimulation of Collagen Production

Type 1 procollagen, the main component of the dermis and a major constituent of the extracellular matrix, plays a key role in the maintenance of skin health. The main factor reducing its synthesis is the activity of extracellular matrix metalloproteinase 1 (MMP–1). Pedunculagin extracted from Mongolian oak (*Quercus mongolica*) increased type 1 procollagen production by 46 and 91% in a concentration-dependent manner (12.5 and 25 µM, respectively) while inhibiting MMP–1 expression [117].

### 8.5. Antimicrobial Activity

The antimicrobial activity of pedunculagin was also evaluated by measuring the diameters of inhibition zones (in mm) during the growth of an isolate of *Staphylococcus aureus* strains. The largest value obtained was 30 mm, with inhibition of the division of methicillin-resistant strains (MRSA) being observed at 13–18 mm. Moreover, pedunculagin reduced the hemolytic activity of *S. aureus* [72].

### 8.6. Antihypertensive Effect

Pedunculagin present in pomegranate fruit extract (*Punica granatum*) inhibits angiotensin-converting enzyme (ACE), with an IC_50_ = 0.91 ± 0.10 µM compared to the positive control captopril (IC_50_ = 0.35 ± 0.01 µM). In addition, it has been proven to act as a competitive inhibitor of ACE with K*_i_* = 1.21 µM, a result which indicates that a relatively low concentration of pedunculagin is required to form the enzyme–inhibitor complex. This finding gives grounds to consider the compound as having antihypertensive properties. In the pathogenesis of hypertension, an increase in oxidative stress is followed by an increase in the production of reactive oxygen species (ROS), which are mediators of vasoconstriction. Strong inhibition of ROS has been demonstrated for pedunculagin (IC_50_ = 0.71 ± 0.11 µM). It is also worth mentioning that one of the main physiological vasodilatory factors responsible for improving blood flow and inhibiting platelet aggregation and adhesion is nitric oxide (NO). This is formed in the endothelium as a result of the activation of endothelial nitric oxide synthase (eNOS) due to the elimination of ROS from the cell or increased levels of intracellular Ca^2+^ [119]. As the levels of pedunculagin increased (concentrations of 2.5, 5, and 10 µM), an increase in intracellular Ca^2+^ was also observed. In addition, pedunculagin used in this concentration range was shown to activate eNOS after 24 h of exposure, and Western blot analysis using anti-eNOS antibodies revealed increased expression of the eNOS protein with respect to the control sample [80].

### 8.7. Hepatoprotective Effect

Pedunculagin at concentrations of 25, 50, and 100 µg/mL induced an in vitro decrease in hepatic enzyme levels of 30%, 32%, and 32% for alanine aminotransferase (ALT) and 45%, 48%, and 48% for aspartate aminotransferase (AST)*,* respectively. The percentage ratios were calculated by comparison with a positive control consisting of liver cells from the HepG2 cell line treated with hepatotoxic carbon tetrachloride (CCl_4_) [65].

### 8.8. Kinase a Inhibition

Kinase A regulates the activity of ion channels. An increase in its activity in the heart results in an accelerated heart rate and increased contraction forces, potentially leading to arrhythmias and heart failure. It has been proven that the galloyl residue present in pedunculagin has an inhibitory effect on the activity of kinase A (ID_50_ = 50 nM) as it inhibits the *β* subunit of kinase A in a dose-dependent manner (ID_50_ = 6.6 nM), whereas the inhibitory effect on the *α* subunit becomes apparent at higher concentrations (ID_50_ = 0.25 μM). This finding provides a basis for classifying pedunculagin as a potent selective inhibitor of the *β* subunit of kinase A, which means it prevents the physiological interaction of the two subunits and inhibits signal transduction at lower concentrations [120].

### 8.9. Atopic Dermatitis Symptom Relief and Skin Protection

Studies on male mice have shown that pedunculagin has a beneficial effect on alleviating the symptoms of atopic dermatitis. To observe these effects, 3 µL of creams containing 0.1% and 0.5% pedunculagin were applied once a day to the backs of mice with previously induced atopic dermatitis. The results showed that there was faster improvement among individuals treated with 0.5% pedunculagin, with less improvement occurring in the group treated with 0.1% pedunculagin and no improvement observed in the untreated mice [121]. In another study, pedunculagin relieved inflammation by enhancing the regeneration of keratinocytes exposed to UVB and also inhibited chemokine and cytokine overexpression induced by UV irradiation in keratinocytes in a concentration-dependent manner. Pedunculagin not only reduced p38, JNK, and ERK phosphorylation in a concentration-dependent manner but also reduced the expression of NF-κB and STAT1, indicating that the compound inhibits the production of chronic inflammatory disease factors induced by UVB irradiation. These results suggest that pedunculagin can be further developed to treat chronic inflammatory skin diseases like atopic dermatitis and psoriasis [122].

### 8.10. SARS-CoV-2 Inhibition

The hydroxyl and ketone groups present in the structure of pedunculagin can bind amino acid residues at the energy point of the 3CL^Pro^ (3–chymotrypsin-like cysteine protease) enzyme of the SARS-CoV-2 virus, resulting in reduced viral replication [123].

### 8.11. Anti-Inflammatory Effect

In order to evaluate the anti-inflammatory effect of pedunculagin, HaCaT cells were treated with lipopolysaccharide (LPS) to induce inflammation. The levels of pro-inflammatory interleukin 6 (IL–6) and interleukin 8 (IL–8) were then measured. Pedunculagin demonstrated anti-inflammatory effects including the inhibition of IL–6 (IC_50_ = 6.59 ± 1.66 μM) and IL–8 (IC_50_ = 0.09 ± 0.41 μM), with the effects observed being stronger than those of epigallocatechin gallate (EGCG) [92].

### 8.12. Topoisomerase II Inhibition

Pedunculagin and 1(*β*)–*O*–galloylpedunculagin were shown to inhibit the activity of topoisomerase II, an enzyme responsible for twisting the DNA helix in the course of replication (IC_100_ = 0.5 µM for both compounds) [124]. As a result, DNA replication and transcription are disrupted, and this can be used to treat cancer.

### 8.13. Inhibition of Protein Phosphatase-1 (PP1) and Protein Phosphatase-2 (PP2)

Ellagitannins represent a novel family of compounds that are able to inhibit PP1 and PP2A distinctly with preferential suppression of PP1. PP1 and PP2A are two major representatives of the phosphoserine/threonine (P-Ser/Thr) specific enzymes and they are believed to be responsible for the dephosphorylation of more than 90% of P-Ser/Thr side chains in cellular phosphoproteins. Several ellagitannins inhibited the activity of protein phosphatase-1 (PP1) and -2 A (PP2A) catalytic subunits (PP1c and PP2Ac) with preferential suppression of PP1c over PP2Ac. The inhibitory potency for PP1c followed the order of tellimagrandin I > mahtabin A > praecoxin B > 1.2-Di-O-galloyl-4.6-(S)-HHDP-β-D-glucopyranose > pedunculagin, with IC_50_ values ranging from 0.20 µM to 2.47 µM. These results establish ellagitannins as partially selective inhibitors of PP1 and indicate that these polyphenols may act distinctly in cellular systems depending on their membrane permeability and/or their actions on cell membranes [125].

The biological effects of pedunculagin are summarized in Table 4.

## 9. Metabolism and Structural Degradation of Pedunculagin

**Figure 2:**
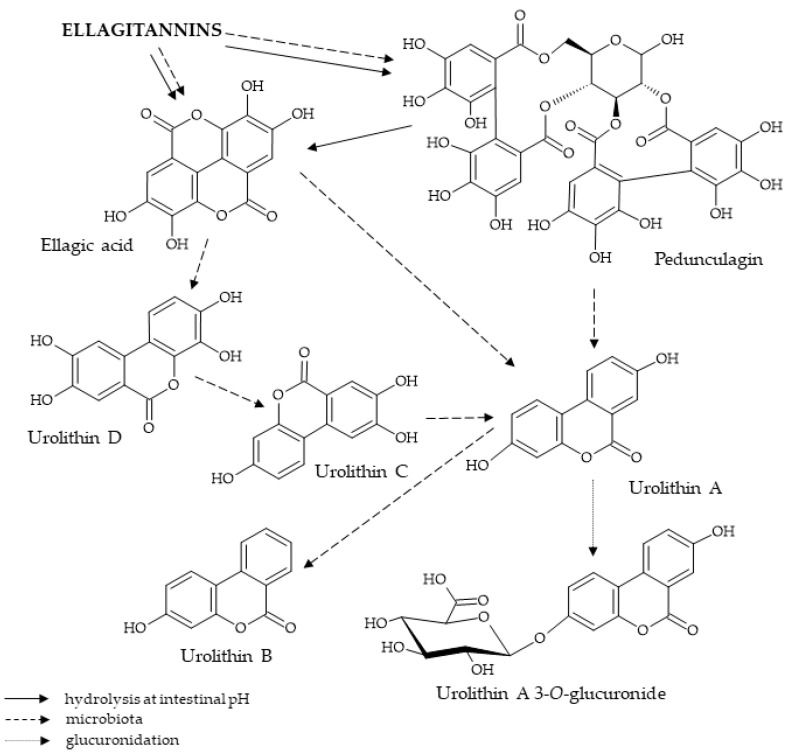
Metabolism of pedunculagin.

Ellagitannins, which include pedunculagin, are hydrolyzed in the acidic environment of gastric acids, resulting in the formation of HHDP acid, which then spontaneously lactonizes to ellagic acid [126]. No presence of ellagitannins in blood and urine has been observed, indicating that they are metabolized by the digestive system. The most common metabolite detected in human urine following the ingestion of raw materials containing ellagitannins is 3,8–dihydroxy–6H–dibenzo[*b,d*]pyran–6–one, known as urolithin B. This is mainly present in the glucuronic acid-conjugated form and, in smaller amounts, as the corresponding aglycone [127]. A study conducted on a group of volunteers involved supplementation with pomegranate juice over five consecutive days and the determination of urinary concentrations of ellagitannin metabolites. In some subjects, low concentrations were observed as early as the first day, with maximum concentrations being reached between days 3 and 4, followed by a slight decrease after 24 h, albeit in significantly lower amounts. On this basis, it was concluded that colonic microbiota were responsible for the metabolism and absorption of the compounds formed by the modification of ellagitannins [128]. Microbiota-mediated degradation of ellagic acid was also suggested by the absence of urolithins in the urine of patients with ileostomies [129]. Figure 2 presents the modifications of the pedunculagin molecule occurring during its systemic metabolism.

Ellagic acid is converted by microorganisms within the intestine through lactone ring cleavage and decarboxylation and dehydroxylation reactions [130]. The first step involves the transformation of ellagic acid to tetrahydroxydibenzopyranone (urolithin D), whose structure includes four phenolic hydroxyl groups. This is followed by gradual detachment of these groups, resulting in the formation of urolithin C, urolithin A, and urolithin B, respectively, as shown in Figure 2.

In order to be converted into more soluble metabolites, urolithins undergo phase II biotransformation, which takes place in the colon wall and liver cells. The resulting glucuronates enter the general circulation and can be excreted in the urine [131]. Due to the involvement of gastrointestinal bacteria in the metabolic process, wide inter-individual variability can be observed in the quantities of products formed [132]. Pedunculagin degradation also occurs in plant organisms. For example, its content in *Camellia japonica* leaves often changes depending on the stage of leaf growth. During the initial stage, when the leaves are soft and young, pedunculagin is a major component, while the pedunculagin content decreases as the leaves thicken with age. This is due to the oxidation reactions that occur in the presence of catechin. Polyphenol oxidase contributes to the conversion of catechin into catechol quinone, which is a strong oxidant. Catechol quinone acts in a non-selective manner on the galloyl rings in the pedunculagin molecule, causing it to oxidize [133].

## 10. Conclusions

Pedunculagin is one of the most abundant and widespread ellagitannins in the plant kingdom. Due to its chemical structure—comprising two HHDP units connected to a glucose core—it serves as a foundational substrate for the biosynthesis of more structurally advanced ellagitannins. As a result, its occurrence is not restricted to any specific taxonomic group. Therefore, when plant materials containing ellagitannins are analyzed, the presence of pedunculagin should be considered. Various biological effects of pedunculagin have been demonstrated in vitro; however, due to the limited availability of the isolated compound, these effects have not yet been directly confirmed in more complex in vivo and clinical models. Nonetheless, its bioactivity can be inferred from studies on plant preparations where pedunculagin is a dominant constituent, suggesting potential health benefits. Further research is required to elucidate the molecular mechanisms of action following topical application as well as the contribution of gut microbiota-derived postbiotic metabolites, urolithins, which are formed after the oral ingestion of preparations containing pedunculagin.

## Figures and Tables

**Table 1:** Pedunculagin-containing plant species.

Genus/Species	Botanical Family	Ref.
*Acca sellowiana* O. Berg (*Feijoa sellowiana* O. Berg)	Myrtaceae	[6,7]
*Alnus glutinosa* L.	Betulaceae	[8]
*Alnus hirsuta* Spach		[9]
*Alnus japonica* Thunb.		[9,10]
*Alnus sieboldiana* Matsum		[9,11,12]
*Agrimonia pilosa* Ledeb.	Rosaceae	[13]
*A. procera* Wallr.		[14]
*Alchemilla mollis* Buser		[15]
*A. viridiflora* Rothm.		[16]
*A. vulgaris* L.		[15,17]
*A. xanthochlora* Rothm.		[18]
*Brachyotum naudinii* Triana	Melastomataceae	[19]
*Bergenia crassifolia* (L.) Fritsch	Saxifragaceae	[20]
*Betula pubescens* Ehrh.	Betulaceae	[21]
*Bredia tuberculata* Guillaumin	Melastomataceae	[22]
*Camellia japonica* L.	Theaceae	[23,24,25]
*C. pachysandra* Hu.		[26]
*Campomanesia phaea* O. Berg.	Myrtaceae	[27]
*Camptotheca acuminata* Decne	Nyssaceae	[28]
*Carica papaya* L.	Caricaceae	[29]
*Carpinus japonica* Blume	Betulaceae	[30]
*C. tschonoskii* Maxim.		[31]
*Castanea mollissima* Blume	Fagaceae	[32]
*Castanopsis fissa* Champ. ex Benth.		[33]
*Casuarina equisetifolia* L.	Casuarinaceae	[34]
*C. stricta* Aiton		[35,36]
*Combretum racemosum* P. Beauv.	Combretaceae	[37]
*Corchorus olitorius* L.	Malvaceae	[38]
*Cytinus hypocistis* (L.) L.	Cytinaceae	[39]
*Duchesnea chrysantha* Zoll. i Moritzi	Rosaceae	[40]
*Eucalyptus* spp. *E. alba* Blume	Myrtaceae	[41]
*E. nitens* H. Deane i Maiden		[42]
*E. viminalis* Labill		[43]
*E. considenina* Maiden		[44]
*Euphorbia makinoi* Hayata	Euphorbiaceae	[45]
*E. prostrata* Aiton		[46]
*E. thymifolia* L. *E. tirucalli* L.		[47]
*Fragaria* spp.	Rosaceae	[48]
*F. × ananassa* Duchesne		[49]
*F. nipponica* Makino		
*Geum aleppicum* Jacq.		[49]
*G. calthifolium* Menzies ex Sm.		[50]
*G. japonicum* Thunb.		[51]
*G. rivale* L.		[52]
*G. urbanum* L.		
*Juglans mandshurica* Maxim.	Juglandaceae	[53]
*J. regia* L.		[54,55,56,57]
*J. sigillata* Dode		[58]
*Kunzea ambigua* Sm.	Myrtaceae	[59]
*Lafoensia pacari* A.St.–Hil.	Lythraceae	[60]
*Lawsonia inermis* L.		[61]
*Liquidambar styraciflua* L.	Altingiaceae	[62]
*Lythrum salicaria* L.	Lythraceae	[63,64]
*Melaleuca styphelioides* Sm.	Myrtaceae	[65]
*Myrciaria cauliflora* Mart.		[66]
*Myrcia multiflora* Lam.		[67,68]
*Paeonia obovata* Maxim.	Paeoniaceae	[69]
*Phyllanthus emblica* L.	Euphorbiaceae	[70]
*Pimenta dioica* L.	Myrtaceae	[71,72]
*Plinia cauliflora* Mart.		[73]
*Platycarya strobilacea* Siebold i Zucc.	Juglandaceae	[74]
*Potentilla* L.	Rosaceae	[75]
*P. anemonifolia* Lehm.		[49]
*P. erecta* L.		[18]
*P. kleiniana* Diels		[13]
*Psidium cattleianum* L.		[35]
*P. guajava* L.		[35,76]
*Punica granatum* L.	Punicaceae	[77,78,79,80,81]
*Rosa laevigata* Michx.	Rosaceae	[82]
*R. roxburghii* Tratt.		[83]
*R. rugosa* Thunb.		[49]
*R. taiwanensis* Nakai		[84]
*R. caesius* L.		[85]
*R. chamaemorus* L.		[5]
*R. coreanus* Miq.		[5]
*R. crataegifolius* Bunge		[49]
*R. fruticosus* L.		[4]
*R. hiraseanus* Makino		[86]
*R. hirsutus* Thunb.		[77]
*R. idaeus* L.		[49,87]
*R. lambertianus* Ser.		
*R. medius* O. Kuntze		
*R. nikaii* Ohwi		
*R. nishimuranus* Koidz.		
*R. occidentalis* L.		
*R. palmatus* Thunb.		
*R. parvifolius* L.		
*R. pheonicolasius* Maxim.		
*R. sanctus* Hruby		
*R. sieboldii* Blume		
*R. trifidus* Thunb.		
*R. utchinesis* Koidz.		
*Quercus acutissima* Carruth.	Fagaceae	[88]
*Q. coccifera* L.		[89,90]
*Q. mongolica* Fisch. ex Ledeb.		[91,92]
*Q. petraea* Vuk.		[93]
*Q. robur* L.		[94]
*Q. serrata* Murray		[95]
*Q. suber* L.		[89,90]
*Quisqualis indica* L.	Combretaceae	[96]
*Sanguisorba officinalis* L.	Rosaceae	[97]
*S. tenuifolia* Fisch. ex Link		[49]
*Sarcopoterium spinosum* L.		[98]
*Sieversia pentapetala* L.		[49]
*Siphoneugena densiflora* O.Berg	Myrtacea	[99]
*Stachyurus praecox* Siebold i Zucc.	Stachyuraceae	[35,36]
*Syzygium aromaticum* L.	Myrtaceae	[35]
*S. aqueum* Burm. f.		
*S. jambos* L.		
*S. samarangense* Blume		
*Terminalia canescens* (DC.) Radlk.	Combretaceae	[100]

**Table 2:** The use of column chromatography (CC) for pedunculagin isolation.

Plant	Plant’s Part	Extract	Column	Mobile Phase	Conditions	Ref.
*Eucalyptus* spp.	leaves	70% EtOH	Diaion HP–20	H_2_O: MeOH	gradual increase in MeOH concentration in H_2_O	[41]
*Lawsonia inermis*	leaves	70% acetone	Diaion HP–20	75% MeOH	not given	[61]
*Rubus caesius*	leaves	EtOAc	polyamide	H_2_O: MeOH	from 1:0 to 0:1 with gradual elution every 10%	[85]

EtOH—ethanol; EtOAc—ethyl acetate; MeOH—methanol.

**Table 3:** The use and conditions of high-performance liquid chromatography (HPLC) for quantitative determination of pedunculagin in plant material.

Plant	Plant’s Part/Sample	Extract	Column	Mobile Phase	Conditions	Content	Ref.
*Duchesnea chrysantha*	herb	80% acetone	µ–Bon–dapak C_18_	ACN: H_2_O (6:4)	not given	653 mg/2.8 kg DW	[40]
*Fragaria* × *ananassa*	roots leaves fruits	not given	Gemini C18 110 Å	ACN: MeOH: H_2_O (63:20:17)	0–5 min, 5% B;	0.43–4.27 mg/g DW	[112]
					5–30 min, 5–28% B;		
					30–40 min, 28–73% B;		
					40–45 min, 73% B;		
					45–47 min, 73–5% B;		
					47–56 min, 5% B.		
*Juglans* *regia*	fruits	75% acetone	Betabasic C18	H_2_O: ACN	0–1 min, 5% B;	not given	[54]
					1–8.5 min, 5–75% B;		
					8.5–9 min, 75–95% B;		
					9–10 min, 95% B.		
*Lawsonia* *inermis*	leaves	50% EtOH	MCI–gel CHP–20P	H_2_O: MeOH (8.5:1.5)	not given	22.9 mg/200 g DW	[61]
*Punica* *granatum*	fruits	juice	HxSil C18	(A) 2% HCOOH in H_2_O (B) 2% HCCOH in MeOH	0–5 min, 10–14% B;	40.4 µmol/L	[113]
					5–16 min, 14–23% B;		
					16–21 min, 23–35% B;		
					21–35 min, 35–40% B;		
					35–38 min, 40–100% B;		
					38–41 min, 100% B isocratic;		
					41–44 min, 100–10% B;		
					44–48 min, 10% B isocratic.		

ACN—acetonitrile; MeOH—methanol; DW—dry weight.

**Table 4:** Summary of the biological effects of pedunculagin.

Activities	Experimental Model	Exposure	Concentration	Efficacy	Ref.
Angiogenic	CAM assay	72 h	15, 30 μg/μL	increase in vascularization and formation of new blood vessels, length and caliber of vessels, number of junctions, and number of complexes	[114]
Anticancer	MCF–7 cell line	48 h in 37 °C	10, 20, 40, 79, 157, 313, 625, 1250 μM	decrease in MCF–7/wt viability at 10 µM—43.7%, at 313 μM 73.3% MCF–7/Adr—not active	[115]
Antioxidant	DPPH in vitro	30 min	not given	IC_50_ = 2.41 ± 0.71 μg/mL	[117]
Collagen production	CCD–986sk cell line	not given	6.25; 12.5; 25 μM	IC_50_ = 9.75 ± 1.29 μM increase in procollagen 1 production: 46% at 12.5 μM and 91% at 25 μM	[117]
Antimicrobial	*S. aureus* strain	24 h in 37 °C	not given	The highest inhibition zone diameter: 30 mm MIC: 312 ± 0 to 2500 ± 0 μg/mL Cell lysis: 36.6% of cells	[72]
Antihypertensive	ACE inhibition	not given	2.5, 5, 10 µM	IC_50_ = 0.91 ± 0.10 µM	[80,119]
Hepatoprotective	CCl_4_–induced hepatotoxicity in HepG2 cells	pretreatment with substance for 1 h	25, 50, 100 µg/mL	Decrease of ALT: 30% at 25 µg/mL, 32% at 50 µg/mL, 32% at 100 µg/mL Decrease of AST: 45% at 25 µg/mL, 48% at 50 µg/mL, 48% at 100 µg/mL	[65]
Kinase A inhibition	phosphorylation of histone H2B	not given	not given	Kinase A: ID_50_ = 50 nM Kinase A subunit *β*: ID_50_ = 6.6 nM Kinase A subunit *α*: ID_50_ = 0.25 μM	[120]
Anti–dermatitis	lesions induced in NC/Nga mice using (TNCB)	before treatment, 1 day, 3 days, 1 week, 2 and 4 weeks afterwards	0.1 and 0.5%	higher concentrations of pedunculagin showed faster and greater improvement	[121]
SARS-CoV-2 inhibition	LigX, MOE–09, catalytic dyad residues of 3CL^pro^ of SARS-CoV-2	–	–	S: –18.58 E–conf: 110.04 E–place: –70.23 E–score: –12.96	[123]
Anti-inflammatory	HaCaT cells	6 h in 37 °C	not given	Inhibition of production: IL–6 (IC_50_ = 6.59 ± 1.66 μM) IL–8 (IC_50_ = 0.09 ± 0.41 μM)	[92]
Topoisomerase II inhibition	topoisomerase II from HeLa cells in the P4 DNA unknotting assay	1 h in 37 °C	not given	pedunculagin IC_100_ = 0.5 µM 1(β)–O–galloylpedunculagin IC_100_ = 0.5 µM	[124]
Protein phosphatases inhibition	HeLa (cervical carcinoma) cells	10 min in 30 °C	0.1–100 µM	pedunculagin PP1c (IC_50_ = 2.47 ± 0.22 μM) PP2Ac (IC_50_ = >100 μM) HeLa Lysate (IC_50_ = 33.90 ± 4.54 μM)	[125]

CAM—chick embryo chorioallantoic membrane; ACE—angiotensin-converting enzyme; ALT—aminotransferase; AST—aspartate aminotransferase; TNCB—2,4,6–trinitrochlorobenzene; S—the final score, which is the score of the last stage that was not set to None; E–conf—the energy of the conformer; E–place—score from the placement stage; E–score—score from the rescoring stage; MIC—minimal inhibitory concentration.
